# A Dimensional Approach to Discrepancy in Parenting Styles in Russian Families

**DOI:** 10.3390/children10081367

**Published:** 2023-08-09

**Authors:** Marina A. Zhukova, Nan Li, Vitalii Zhukov, Elena L. Grigorenko

**Affiliations:** 1 Department of Psychology, University of Houston, Houston, TX 77204, USA; marina.zhukova@times.uh.edu (M.A.Z.); nan.li@times.uh.edu (N.L.); 2 Center for Cognitive Sciences, Sirius University of Science and Technology, Sochi 354340, Russia; 3 Texas Institute for Measurement, Evaluation, and Statistics, University of Houston, 4349 Martin Luther King Boulevard, Houston, TX 77030, USA; 4 Department of Computer Science, University of Houston, Houston, TX 77204, USA; vzhukov@uh.edu; 5 Department of Molecular and Human Genetics, Baylor College of Medicine, Houston, TX 77030, USA; 6 Child Study Center, Yale University, New Haven, CT 06520, USA

**Keywords:** parenting styles, warmth, demandingness, mental health, CBCL

## Abstract

We investigated the magnitude and direction of differences in parenting styles as they relate to children’s mental health problems, as assessed using the CBCL. The sample consisted of 306 families residing in a large industrial city in Russia. We aimed to expand the cross-cultural literature on parenting styles by assessing a sample of Russian families and analyzing how agreement versus disagreement between self-reported and partner-reported parenting styles related to children’s mental health problems. The findings suggested that both congruence and incongruence between parenting styles could be associated with children’s mental health problems. When parents agreed about high warmth and matched on lower levels of demandingness, in line with the permissive parenting style, children tended to exhibit maladaptive behavior and externalizing problems. We also registered that children were likely to show low levels of mental health problems when fathers had higher self-reported warmth compared with mothers’ reports. In contrast, children whose fathers had higher self-reported demandingness compared with the mothers’ reports, exhibited moderate levels of mental health problems. This study expands the existing literature by providing a dimensional approach to children’s mental health difficulties in the context of (dis)agreements in the parenting styles within a family.

## 1. Introduction

Parenting styles refer to a set of emotional and behavioral practices that have been linked to children’s mental health, academic achievement, adaptive functioning, delinquency, and interpersonal relationships. The differences in parenting styles have been widely studied in the context of Baumrind’s model [1], which introduced a two-dimensional framework of warmth/responsiveness and demandingness/control. Based on this model, Maccoby and Martin [2] suggested that all parenting styles fall in one of the four quadrants depending on the expression of warmth/responsiveness and demandingness/control and are classified as authoritative parenting (high warmth and high demandingness), authoritarian parenting (low warmth and high demandingness), indulgent parenting (high warmth and low demandingness), and neglectful parenting style (low warmth and low demandingness). Parenting styles are often assessed by relying on self-reports from one or both parents, where these reports represent the parents’ perceived goals and behaviors in raising their children. Current literature is dominated by mothers’ self-reports, providing limited information about differences in parenting within a family and how they relate to children’s developmental outcomes. Discrepancies between parents have rarely been studied, with existing research often treating mothers and fathers equivalently by either having only the mother fill out the questionnaires or averaging responses across both parents. Yet, studies have demonstrated that greater disagreement in parenting styles is associated with an increased risk of mental health problems and behavioral problems in children [3,4]. While the direction of the differences between the parents remains inconclusive, studies have suggested that disagreements in parenting styles play an important role in explaining children’s developmental outcomes above and beyond the contributions made by an individual parent [5,6].

The literature on parenting styles is dominated by research on white middle-class families in the United States (US). Cross-cultural studies questioned the generalizability of the findings derived from those samples, highlighting the importance of studying samples from diverse backgrounds [6,7,8]. Therefore, the first goal of the present study was to expand the cross-cultural literature on parenting styles by assessing a sample of Russian families and analyzing how agreement versus disagreement between self-reported and partner-reported parenting styles related to children’s mental health. 

Second, most studies pertinent to disagreements in parenting styles utilized a categorical approach by classifying parenting styles as either authoritative, authoritarian, indulgent, or neglectful. This approach has been criticized in the field, as it limits the spectrum of parenting behaviors given that parents rarely endorse only one particular style [9]. Thus, the second goal of the present study was to address this methodological shortcoming by evaluating the disagreements in parenting styles between mothers and fathers on the dimensions of warmth and demandingness in line with the model proposed by Maccoby and Martin [2].

Finally, previous studies that linked disagreements in parenting styles to children’s mental health utilized the categorical approach to behavioral problems, which failed to address the issue of dimensionality of behavioral issues. Given a high rate of comorbidity between internalizing and externalizing symptoms [10], it is crucial to address a noteworthy methodological concern by shifting the focus of analyses away from distinct clinical scales. The current study addressed this issue of homotypic (within internalizing or externalizing spectrum) and heterotypic (across internalizing and externalizing spectra) comorbidity of mental health by investigating the latent profiles of children’s mental health problems and their relationship to parental disagreements between self and partner reports and their dissimilarities in childrearing practices.

### 1.1. Discrepancies in Parenting Styles

The assessment of parenting styles is typically indirect by relying on parental self-reports or child reports. Previous studies registered a discrepancy between the parent’s and children’s perceptions of parenting styles [11], where the magnitude of disagreements depended on the measured facet of parenting style and the child’s age. Discrepancies are typically larger for adolescents compared with younger children and their parents. In general, children tend to overreport negative aspects of parenting, while parents tend to underreport problematic practices by providing a more favorable perspective. A meta-analysis demonstrated that both mothers and fathers rated themselves higher on warmth and acceptance compared with their children [12]. These discrepancies are often viewed as a sign of a family conflict [13], while other researchers argue that low agreement with parents is developmentally appropriate for adolescents [14]. Obtaining reports from children and parents has become a standard practice in research on family functioning [12]. However, most studies on child–parent discrepancies have overlooked the potential disagreements between parents. Mothers typically serve as the only informants by providing reports on self and partner’s parenting styles [15]. The use of only maternal reports presents a significant bias that dominates the current literature on parenting styles. The importance of the father’s involvement in parenting and its impact on children’s mental health, social functioning, and academic achievement has been well-documented. For instance, Martin and Ryan [16] showed that paternal supportiveness could compensate for the lack of maternal support and lead to increased child competence. Roopnarine and Krishnakumar [17] found that fathers’ authoritativeness in a sample of Caribbean immigrants in the US was related to higher levels of social and communicative skills in toddlers compared with mothers’ parenting styles. These studies emphasize the importance of accounting for the parenting styles of both parents and their links to children’s development.

Only a few studies to date have investigated differences in parenting styles between mothers and fathers and their contributions to the child’s mental health problems and student engagement. A longitudinal study that used observational data and children’s reports of parenting behaviors found a high degree of similarity between the parenting styles of mothers and fathers [4]. The findings showed that a combination of two authoritative parents or an authoritative and an indulgent parent was linked to the best outcomes in children. In particular, an authoritative mother combined with an indulgent father was identified as an optimal combination of approaches for preventing child delinquency and depression, while the combination of two authoritative parents was linked to the highest level of school commitment in children. The worst child outcomes were observed in families with uninvolved parents or combinations of uninvolved and indulgent parents [4]. These findings are in line with the literature that identified the authoritative parenting style as an optimal one. Gamble and Ramakumar [6] found a small but positive correlation between mothers’ and fathers’ self-reported parenting styles (*r* = 0.26), also pointing to a similarity in parenting approaches but only on the dimension of authoritativeness.

A study by Rinaldi and Howe [18] conducted on a sample of 59 primarily white families in the US showed that a combination of maternal permissiveness and paternal authoritarian style was associated with externalizing problems in toddlers. This study pointed to the detrimental effects of conflicting parenting approaches. Braza and Carreras [19] demonstrated that a combination of an authoritarian maternal style and a permissive paternal style was associated with aggressive behavior in children of both genders and was negatively associated with internalizing problems in boys only, suggesting that the combination of parenting styles can have a differential effect on children depending on gender. 

Notably, previous studies rarely considered the direction of differences in parenting styles and their relationship with children’s outcomes. The magnitude of differences and their meaning for developmental outcomes has been overlooked. Tavassolie and Dudding [20] found that a greater difference in parents’ self-perceived permissiveness was related to a higher rate of child externalizing problems. In families where mothers viewed themselves as more authoritative compared with fathers, children had a higher rate of behavioral problems and increased internalizing problems. However, when fathers rated themselves as more permissive than mothers, children demonstrated fewer internalizing and overall behavioral problems. While the observations about the direction and magnitude of the differences are inconclusive, the literature suggests that families with similar parenting styles are characterized by better children’s outcomes. From a family systems perspective, these can be explained by higher cohesion within the family.

### 1.2. Cross-Cultural Differences in Parenting Styles

Early studies has suggested that authoritative parenting style is an optimal parenting approach [21,22]. It is associated with enhanced academic achievement and reduced mental health problems in children [23]. In contrast, neglectful, indulgent, and authoritarian parenting styles have been linked to behavioral problems and poor achievement, with the neglectful parenting style being the most detrimental [24]. However, most existing studies were conducted among white middle-class families from the US, which raised concerns about the cultural sensitivity of the findings. Overall, cross-cultural research on parenting styles suggests that an authoritative style might not adequately reflect the socialization goals and cultural values of non-Western societies [25]. A recent meta-analysis [25] investigated whether the effects of parenting styles on academic achievement and child well-being would differ based on the collectivistic or individualistic orientation of the culture. Contrary to the authors’ expectations, the findings demonstrated more similarities in parenting practices across the globe. Authoritarian parenting was associated with more internalizing and externalizing problems and lower academic achievement in both individualist and collectivist cultures. Research indicates that parents in post-communist countries exhibit high control in their parenting practices [26], which is often linked to the values of collectivism [27].

### 1.3. Parenting Styles in the Russian Culture

In general, cross-cultural research that includes Russian samples either focuses on immigrant families or merges Russian samples into a larger group of Eastern Europeans. Russia is described as a collectivist society based on Hofstede’s model, scoring 39 out of 100 points on the collectivism–individualism scale, where higher scores indicate individualism [28]. Correspondingly, researchers typically expect to observe a higher prevalence of authoritarian parenting [25], but this theoretical expectation is not supported by the literature. Grigorenko and Sternberg [29] showed that according to parents’ self-reports, the most common parenting styles in Russian families were authoritative (33%) and neglectful (33%), followed by authoritarian (22%) and indulgent/neglectful (12%). The study also showed that better family cohesion was observed in authoritative and indulgent parenting styles. Importantly, child outcomes and their relation to parenting styles were not directly investigated in the study. In a cross-cultural study, it was discovered that Romanian and Russian adolescents reported higher rates of authoritative parenting and lower rates of authoritarian parenting style compared with their French counterparts, which contradicted the initial expectations [30]. These findings are consistent with a study showing that Russian children perceive their parents as more authoritative than authoritarian [31]. According to Glendinning [31], the optimal combination of reduced control and high warmth, which refers to indulgent parenting, is associated with higher adjustment in a diverse sample of Siberian adolescents. A study by Hart and colleagues [32] demonstrated that low family cohesiveness, parental disagreements, and an authoritarian style characterized by coercive discipline were linked to higher aggression in Russian children. At the same time, higher levels of paternal responsiveness were associated with less overt aggression. Parental warmth in an authoritative parenting style served as a protective factor against aggression, especially for males, as demonstrated in a longitudinal study [33]. These results are consistent with studies conducted in the US. The major cultural finding that did not translate to research on Western samples is that parental level of education was not related to aggression.

A brief literature review suggests that the evidence about the cross-cultural appropriateness and effectiveness of specific parenting styles remains inconclusive. Russia is often considered a collectivist society, giving credit to its Soviet past, but it might not reflect its current orientation. The present study aimed to address the scarcity of data obtained from Russian samples and the lack of consistency in findings about the effectiveness of parenting styles by analyzing a representative sample of Russian families. This study further expanded the literature on post-Soviet countries, which blend the Eastern and Western values that can shape beliefs about parenting [34].

### 1.4. Dimensional Approach to Children’s Mental Health Problems

Parenting styles are often studied in relation to children’s mental health problems. In this line of research, children’s behavioral problems serve as a metric of the impact and efficacy of parenting strategies. Indicators of children’s mental health problems have been used as outcome variables in studies analyzing optimal parenting styles [8,23], cross-cultural differences in parenting [21,35], and discrepancies in parenting styles [3,13]. A gold standard method to identify mental health and behavioral problems in children is The Achenbach System of Empirically Based Assessment (ASEBA). ASEBA has three versions: youth self-report [36], parent report [37], and teacher report [38]. ASEBA utilizes a dimensional approach by organizing behavioral problems in syndromes that allow for capturing problems on the internalizing and externalizing spectra. This approach reflects the methodology of developmental psychopathology, which accounts for the comorbidity of symptoms. Comorbidity refers to the co-occurrence of multiple disorders, which can either fall in the same diagnostic grouping (homotypic comorbidity) or cut across diagnostic groups (heterotypic comorbidity) [10].

Examples of homotypic comorbidity in children include anxiety and depression, ADHD, and delinquency. Heterotypic comorbidity occurs in cases of substance abuse and anxiety, conduct disorder with depression. Studies show that both internalizing and externalizing [36] behaviors are best conceptualized in dimensional rather than categorical terms. Wadsworth and Hudziak [39] showed that children in clinical and non-referred samples differ in the severity of symptoms but do not show exclusively depressive or anxious symptoms. There is a high degree of co-occurrence of syndromes that are evaluated using ASEBA ranging from 11.0% to 35.5% in the general population [40]. The absence of pure classes of disorders suggests that they should be studied using latent profile modeling. A recent study demonstrated that factor mixture modeling (FMM), which allows for accounting not only for multiple co-occurrences of syndromes but also to incorporate their severity, fits both the data from parental (CBCL) and teachers’ reports (TRF) [41]. However, this approach has not been routinely used in studies where children’s mental health serves as the outcome variable in the assessment of parenting styles. Those assessments typically include internalizing, externalizing, and total behavioral problem scales, while failing to account for the overlap in symptoms. The rationale of the current study was to identify whether disagreements between self- and partner-perceived parenting styles in childrearing practices were associated with children’s membership in distinct classes of mental health problems. The latent profile analysis of children’s outcomes allowed us to account for multiple syndrome co-occurrences in line with previous studies that identified distinct groups using the CBCL based on syndrome co-occurrence and symptom severity [41].

The present study focused on the following research questions: (1) Are there discrepancies in how parents perceive their own and their partners’ parenting styles? (2) Are there distinct latent groups of mental health problems in children based on parents’ (CBCL) reports? (3) Are memberships in latent mental health problem profiles related to the discrepancy between mothers’ and fathers’ perceived parenting styles? (4) Does the magnitude and the direction of disagreements between perceived parenting styles affect children’s mental health problems? We hypothesized that the discrepancy between self-perceived and partner-perceived indices of parenting styles operationalized as parental warmth and demandingness would be associated with the presence and severity of mental health problems in children. The current study expands the existing literature by providing a cross-cultural perspective on self- and partner-perceived differences in parenting styles, viewing parenting styles dimensionally instead of assigning discreet categories, and by identifying latent profiles of mental health difficulties in children as they relate to parenting styles.

## 2. Materials and Methods

### 2.1. Participants

The data were collected between 1999 and 2000 in Voronezh, an industrial city in central Russia, where the population is reasonably representative of other urban regions of Russia in terms of socioeconomic status (SES) and other demographic characteristics, such as income and education [29]. Voronezh, being a regional center, provides insights into parenting patterns that are more representative compared with metropolitan regions in Russia (such as Moscow). Additionally, children typically attend the same public schools irrespective of their family income due to the scarcity of private schools in the area. A total of 489 mothers and 327 fathers were recruited for the study. Parents were recruited from schools that their children attended. All families participating in the study were Russian-speaking; however, information about their ethnic and cultural identity was not collected. They were invited to come to the school over the course of two months and were asked to fill out questionnaires and participate in the testing. When parents had multiple children, data from only one child (chosen randomly by the researchers) was included in the study. The parents received financial compensation for their participation in the study. The study was conducted in accordance with the Declaration of Helsinki and approved by the Institutional Review Board of Yale Child Study Center, project identification code 0409000096.

As the main focus of the study was the similarity and discrepancy between maternal and paternal parenting styles, data from intact families (where both parents were present) were used for the study. Data from families with other primary caregivers (i.e., grandparents) were excluded from the analysis. The studied sample consisted of 306 intact families, where both parents reported being married, cohabiting with their partner, and raising children together: 306 mothers and 306 fathers with children (54.2% girls, 45.8% boys) in the age range of 8–17 years (*M* = 12.59, *SD* = 2.84). When parents had multiple children, data from only one child (chosen randomly by the researchers) was included in the study. In terms of educational status for mothers, 1 (0.3%) did not have a high school diploma, 47 (15.4%) graduated from high school, 75 (24.5%) completed vocational training, 5 (1.6%) had unfinished vocational education, and 167 (54.6%) had a college degree; for fathers, 2 (0.7%) did not have a high school diploma, 57 (18.6%) graduated from high school, 78 (24.5%) graduated from vocational schools, 8 (2.6%) had unfinished vocational education, and 148 (48.4%) had a college degree. There were no significant differences in the educational statuses of mothers and fathers (*t* (587) = −1.80, *p* = 0.07).

### 2.2. Measures

#### 2.2.1. Beliefs about Parenting Styles

To evaluate parenting styles, data were collected through self-reports and partner reports from both mothers and fathers. Each parent answered the questions about their own parenting styles (mother self-reported and father self-reported parenting styles) and then answered the same set of questions about their partners (mothers’ perception of their partners’ parenting styles and fathers’ perception of their partners’ parenting styles). The questionnaire included 37 Likert scale items, with responses ranging from 1 (completely disagree) to 9 (completely agree). A composite score was created by summing the responses of all items for each participant, ranging from 37 to 333. The questions included general beliefs about parenting, like “I am confident I know what is best for my child”, “I would feel fine buying a doll for my son and a toy truck for my daughter”, and “Children should be allowed to express both positive and negative emotions”. The questionnaire provides results on parenting styles across two dimensions: warmth and demandingness, in line with the model proposed by Maccoby and Martin [2]. The measure was used in previous research in Russia, and the psychometric characteristics of the scale, as well as item examples, were described in Grigorenko and Sternberg [29]. The split-half reliability coefficients for both scales were high: α = 0.82 for the warmth scale and α = 0.78 for the demandingness scale.

#### 2.2.2. Children’s Mental Health Problems

A Russian version of a well-validated system of the Achenbach System of Empirically Based Assessment (ASEBA) was used [42] to assess child mental health problems. ASEBA is a widely used comprehensive approach for evaluating the behavioral, emotional, and social functioning of children and adolescents and provides a standardized method to assess various aspects of psychopathology and adaptive functioning. The ASEBA comprises a set of questionnaires and rating scales that are completed by different informants, including parents, teachers, and the adolescents themselves. In the current study, children’s mental health data were obtained from parental reports (CBCL [37]). ASEBA was translated into Russian and showed good psychometric properties, with Cronbach’s alpha ranging between 0.90 and 0.97 for parents and between 0.88 and 0.97 for teachers [43,44,45]. The Russian CBCL questionnaire retains the structure and content of the original instrument while ensuring that the language and cultural context are relevant and appropriate for the target population. The CBCL is comprised of eleven subscales: eight individual subscales measure delinquent behavior, aggressive behaviors, withdrawal, somatic complaints, anxiety/depression, social problems, thought problems, and attention problems. Three composite scales reflect externalizing problems (comprises delinquent and aggressive behaviors), internalizing problems (comprises withdrawal, somatic complaints, and anxiety/depression problems), and total problems (includes externalizing, internalizing, social, thought, and attention problems). All questionnaires include a set of behavioral characteristics of a child that are rated from 0 (not true) to 2 (very true or often true). Greater scores indicate higher severity of mental health symptoms. We converted the raw scores for the eight subscales into *T*-scores to facilitate the interpretation of the subscale scores.

### 2.3. Analytic Plan

#### 2.3.1. Traditional Approach Using Difference Scores

In line with previous research [20,46], we calculated mother–father discrepancy scores on parenting styles in four ways. The first two approaches were based on the self-report and partner-perceived scores of parenting styles. First, we subtracted the father’s perception of his partner’s warmth (FPW) from the mother’s self-reported warmth (MW) to capture the discrepancy in warmth for mothers (MW − FPW); we subtracted the father’s perception of demandingness (FPD) from the mother’s self-report of demandingness (MD) to capture the discrepancy in demandingness for mothers (MD − FPD). In a similar way, we calculated the discrepancy scores of warmth (father’s self-reported warmth (FW) − mother’s perception of her partner’s warmth (MPW)) and demandingness (father’s self-reported warmth (FD) − mother’s perception of her partner’s warmth (MPD)) for fathers. These discrepancy scores were sensitive to the direction of disagreement and the magnitude of disagreement, such that positive scores indicated that parents overreported on the given dimension of their parenting style relative to their partner’s perception, and negative scores indicated that parents underestimated their expression of warmth/demandingness relative to their partner’s perception of their parenting styles. Second, we calculated absolute differences for the four aforementioned discrepancy scores based on the self-reported and perceived scores of parenting styles: |MW − FPW|, |MD − FPD|, |FW − MPW|, and |FD − MPD|. The absolute discrepancy scores gauged the magnitude of disagreement in the mother–father dyads. The other two approaches were based on self-report scores of parenting styles. Third, we calculated the raw discrepancy scores using the mother’s and father’s self-reported scores: MW − FW and MD − FD. Lastly, we took the absolute value of the self-reported discrepancy scores to obtain |MW − FW| and |MD − FD|. Bivariate correlations and descriptive statistics related to parenting styles were calculated using R [47].

A three-step latent profile analysis (LPA) [48] was conducted to identify distinct subgroups of children with similar patterns of mental health problems and to examine the associations between these subgroups and the discrepancy in parenting styles. Regular LPA was used to identify distinct subgroups based on the eight symptoms of child mental health problems: anxiety/depression, withdrawal, somatic complaints, social problems, thought problems, attention problems, delinquent behavior, and aggressive behavior. A variety of latent profile solutions (1–4 latent profiles) were evaluated to identify the optimal solution. To identify the optimal number of latent profiles in each model, we examined the Akaike information criterion (AIC), the Bayesian information criterion (BIC), sample-size-adjusted BIC (SSABIC), entropy, the Vuong–Lo–Mendell–Rubin adjusted likelihood ratio test (VLMR-LRT), the bootstrapped likelihood ratio test (BLRT), the proportion of respondents in each profile, and the theoretical interpretation. Smaller AIC, BIC, and SSABIC values indicate a better fit of the model. The VLMR-LRT and BLRT provide a *p*-value, which indicates whether the *k* − 1 profile model can be rejected in favor of the *k* profile model, with *k* being the number of classes. It was suggested that an entropy value greater than 0.80 indicates that the derived latent profiles are highly discriminating [49]. Finally, the proportions of individuals in each class were presented. In order to identify empirically relevant classes, we focused on the classes containing greater than 10% of the sample (greater than 30 participants).

After identifying the optimal model for the CBCL, we proceeded with including auxiliary variables to examine their relationships with the membership of the retained LPA models. Covariates, including child gender and age, were common across all models. Six prediction models were developed for the CBCL. The primary predictors were |FW − MPW| and |FD − MPD| for model 1, FW − MPW and FD − MPD for model 2, |MW − FPW| and |MD − FPD| for model 3, MW − FPW and MD − FPD for model 4, |MW − FW| and |MD − FD| for model 5, and MW − FW and MD − FD for model 6. 

Mplus Version 8.0 [50] was employed to examine the models, and the R3STEP function in Mplus was used for the prediction of membership of an LPA model. One participant had missing data on all eight individual subscales of CBCL, and thus, this participant was excluded from the analysis. Another participant had one missing data point on the somatic complaints subscale for CBCL. Full information maximum likelihood (FIML) estimation was used to handle missing data.

#### 2.3.2. RSA Analytic Approach

To further examine the discrepancy between parents’ self-reports and partner reports, as well as the differences between self-reports in parenting styles, a response surface analysis (RSA) was utilized. RSA presents the results of the polynomial regression analyses in a three-dimensional space, which allowed us to study a more nuanced relationship between the two predictors as opposed to deriving a difference score between them [51]. RSA emerged in response to shortcomings associated with the use of the difference scores [52]. For instance, difference scores are not able to determine the extent to which each of the variables contributed to the outcome. Therefore, various sets of differences can lead to the same average difference score when not accounting for the contribution, direction, and extent of differences between predictors [53]. RSA overcomes these limitations by testing the congruence hypothesis. This technique allowed us to examine whether the degree of congruence between the two variables affected the outcome variable. RSA is a robust method that can test hypotheses of curvilinear effect and moderation without using the interaction term of independent variables. The coefficients of the polynomial regression equation are used to examine the response surface pattern, which is a graph that provides a three-dimensional visual representation of the data [53]. The slope and curvature of two slopes of predictors, namely, the line of congruence (LOC) and the line of incongruence (LOIC), represent possible response surface patterns [54]. The LOC indicates how the level of congruence between two predictor variables (X and Y) affects the outcome variable (Z). It examines the effect of congruence on the outcome across the continuum of two predictors. This approach allowed us to explore all possible combinations of X = Y and determine whether the effects differed at low, intermediate, and high scores of the predictors. The incongruence line (LOIC) covered all valid combinations of predictors for which Y = −X. It captured how the degree of discrepancy between the two predictors affected the outcome variable. Summing up, the LOC refers to the numerical congruence of the scales, while the LOIC corresponds to numerical differences in one or the other direction. This numerical comparison can be done when two variables are measured on the same scale. RSA is divided into two stages described in Rodrigues (2021): the first step involves the estimation of a second-order polynomial regression: (*Z = b*_0_ *+ b*_1_*X + b*_2_*Y + b*_3_*X*^2^ *+ b*_4_*XY + b*_5_*Y*^2^ *+ e*), where *Z* indicates a dependent variable, *X* is predictor 1, and *Y* is predictor 2. Therefore, the outcome variable *Z* is regressed against its predictors *X* and *Y*, their respective square terms *X*^2^ and *Y*^2^, and their interaction *XY*. The second step entails using the results of the model to generate a response surface and analyzing the importance of the effects.

To implement the RSA approach, we transformed the data into the wide format, where each row contained data from one parental dyad. Mothers’ and fathers’ self-reports and partner reports on warmth and demandingness were used as predictor variables. Children’s behavioral and emotional problems indices measured with the CBCL were used as outcome variables: separate models were created using the total problem behavior index and the internalizing and externalizing problem scales, as the RSA does not allow for testing multivariate hypotheses. Next, predictor variables were standardized using grand mean centering. The RSA was completed in R using the RSA package [55]. 

## 3. Results

### 3.1. Descriptive Statistics for Parenting Styles

Means, standard deviations, and Pearson correlations for the raw discrepancy scores and the absolute discrepancy scores are presented in Table 1. Mothers tended to underestimate their warmth (MW *−* FPW = −4.38) but overestimate their demandingness compared to fathers’ reports (MD *−* FPD = 3.64). Although fathers tended to underestimate their warmth and demandingness, their self-perceived parenting styles were close to how their partners perceived their parenting styles (FW *−* MPW = −1.79 and FD *−* MPD = −0.81). The correlation between warmth and demandingness was 0.20 for mothers and 0.12 for fathers for the raw discrepancy scores. The correlation between warmth and demandingness was 0.31 for mothers and 0.37 for fathers for the absolute discrepancy scores. On average, the mother’s self-reported warmth was lower than the father’s self-reported warmth (MW *−* FW = −6.09), whereas the mother’s self-reported demandingness was higher than the father’s self-reported demandingness (MD *−* FD = 5.93). 

### 3.2. Latent Profiles of Child Behavioral Problems

**Figure 1:**
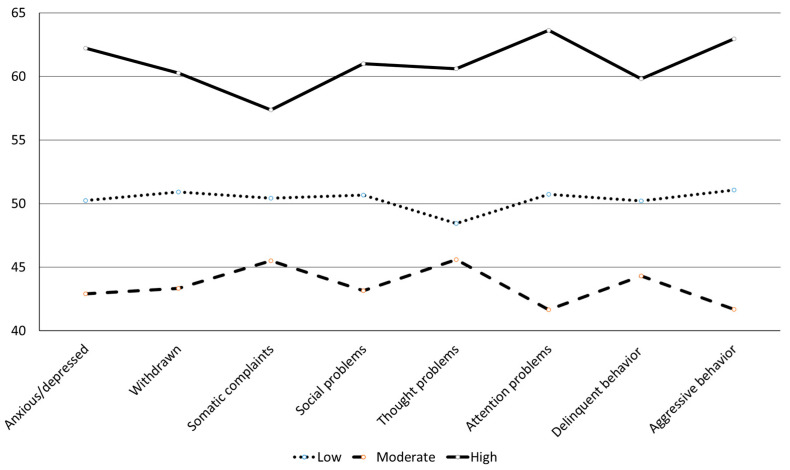
Line Graph Comparing Profiles on Indicator Variables in the *T*-score Format for the CBCL.

Table 2 presents model fit statistics for LPA. For the CBCL, AIC, BIC, and SSBIC decreased from one profile to four profiles. The four-profile solution produced one profile with a relatively small sample size (*n* = 14, around 5% of the entire sample), suggesting potential over-extraction. The VLMR-LRT and BLRT were significant when comparing the three-profile solution with the two-profile solution, suggesting that the three-profile solution was favorable. Therefore, the three-profile solution was retained. The three profiles (subgroups) were characterized as follows (Figure 1). Profile 1 (*n* = 121, 40%) was characterized by children whose parents rated them as having symptoms on all eight CBCL domains below the mean, which was referred to as the low-symptom-severity profile. Profile 2 (*n* = 119, 39%) was characterized by children whose parents rated them as having symptoms for the eight CBCL domains around the sample mean, which was referred to as the moderate-symptom-severity profile. Profile 3 (*n* = 65, 21%) was characterized by children whose parents rated them as having symptoms for the eight CBCL domains above the mean, which was referred to as the high-symptom-severity profile.

The identified classes reflected a high degree of comorbidity between the symptoms and behavioral problems, indicating that the individual profiles were best distinguished based on the severity of the symptoms as opposed to a specific constellation of symptoms (e.g., syndromes). The observed pattern indicates the importance of utilizing individual scales rather than broad behavior scales (e.g., internalizing and externalizing), as they provide a more nuanced approach to the analysis of mental health problems in children.

### 3.3. Latent Profile Analyses with Discrepancy Scores of Parenting Styles

For the CBCL, child gender, age, and a variety of discrepancy scores of parenting styles were included as covariates in the three-profile solution (Table 3). The low-severity symptom profile was considered the reference group. Gender and age were not related to the likelihood of being in the low-severity symptom profile versus the moderate-severity symptom profile, but they were related to the likelihood of being in the low-severity symptom profile versus the high-severity symptom profile. Specifically, girls were less likely to be members of the high-severity symptom profile than boys (OR = 0.48 to 0.50). Older children were less likely to be members of the high-severity symptom profile than younger children (OR = 0.85 to 0.86). Results of Model 1 showed that children with higher absolute discrepancy scores between fathers’ self-reported warmth and mothers’ perception of their partners’ warmth (|FW *−* MPW|) were more likely to be members of the high-severity symptom profile (OR = 1.04). The results of Model 2 indicate that the raw discrepancy scores between fathers’ self-reported demandingness and mothers’ perception of their partners’ demandingness (FD *−* MPD) were related to a higher likelihood for the child of being a member of the moderate-severity symptom profile controlling for child gender and age (OR = 1.04). The results of Model 2 also show that children with a higher raw discrepancy score between fathers’ self-reported warmth and mothers’ perception of their partner’s warmth (FW *−* MPW) were less likely to be in the high-severity symptom profile (OR = 0.96). Neither the raw (MW *−* FW and MD *−* FD) nor the absolute (|MW *−* FW| and |MD *−* FD|) self-reported discrepancy scores of parenting styles were associated with the mental health profiles for the CBCL.

### 3.4. Response Surface Analysis 

**Figure 2:**
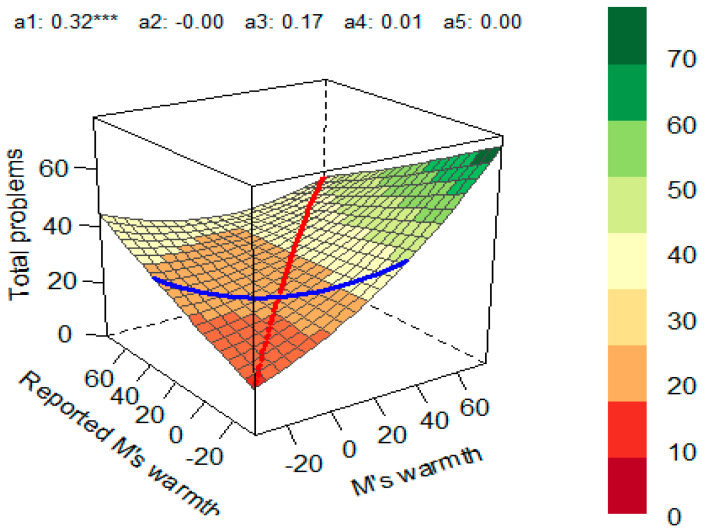
Response Surface for the Impact of Mothers’ Self-Reported Warmth and Fathers’ Perception of Maternal Warmth on Total Behavioral Problems in Children. Red line—line of congruence; blue line—line of incongruence (LOIC); *a*_1_—slope symmetry line (linear additive effect on the line of congruence); *a*_2_—curvature symmetry line (curvature on the line of congruence); *a*_3_—slope asymmetry line (ridge shifted away from the LOC); *a*_4_—curvature asymmetry (line curvature on the line of incongruence); *a*_5_—the interaction coefficient between the first and second predictor variables. *** *p* < 0.001.

Our next goal was to examine whether the direction and magnitude of the discrepancy in parental self-perceptions and their partners’ reports of their parenting styles could predict children’s mental health problems. First, the dimension of mothers’ warmth was examined. We inspected the number of dyads that had discrepancies between the two predictors. The percentage of congruence (agreement) was 58%. In 12% of dyads, mothers reported higher warmth compared with how fathers rated them, and in 30% of dyads, mothers indicated lower warmth compared with fathers’ perception of maternal warmth. Given the meaningful presence of the discrepancy in predictors, a polynomial regression was run to predict the total behavioral problems using the congruence between parental perceptions of warmth. The polynomial model was significant (*R*^2^ = 0.067, *p* < 0.001). Next, the coefficients and the response surface were analyzed. The linear additive effect on the line of congruence was significant (*a*_1_ = 0.32, *p* = 0.007), suggesting that the total behavioral problems of children were higher when mothers’ self-reported warmth and fathers’ perception of maternal warmth matched at high levels (Figure 2). The effect was further followed up using internalizing and externalizing scores as the outcome variables. While the model for internalizing problems did not yield significance (*R*^2^ = 0.018, *p* = 0.355), the polynomial model for externalizing problems was significant (*R*^2^ = 0.01, *p* < 0.001). The linear additive effect on the line of congruence (*a*_1_ = 0.14, *p* < 0.001) suggested that externalizing problems were higher when mothers’ and fathers’ perceptions of maternal warmth matched at higher levels than at lower levels. 

**Figure 3:**
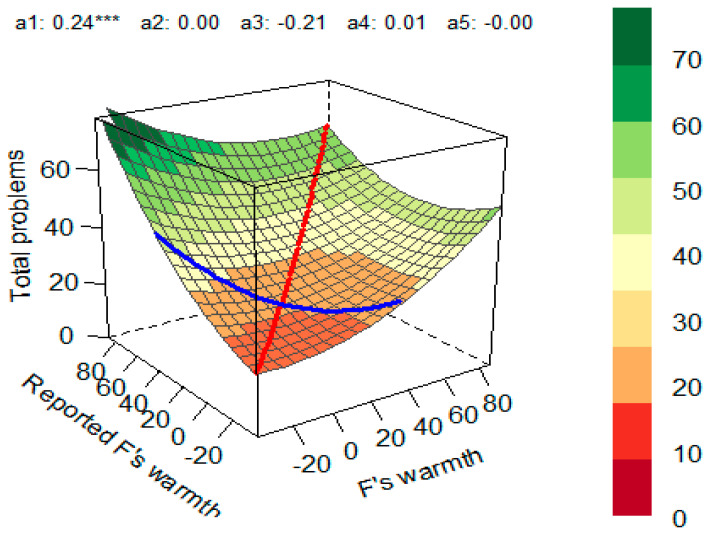
Response Surface for the Impact of Fathers’ Self-Reported Warmth and Mothers’ Perception of Paternal Warmth on Total Behavioral Problems in Children. Red line—line of congruence; blue line—line of incongruence (LOIC); *a*_1_—slope symmetry line (linear additive effect on the line of congruence); *a*_2_—curvature symmetry line (curvature on the line of congruence); *a*_3_—slope asymmetry line (ridge shifted away from the LOC); *a*_4_—curvature asymmetry (line curvature on the line of incongruence); *a*_5_—the interaction coefficient between the first and second predictor variables. *** *p* < 0.001.

Next, the congruence between fathers’ self-reported warmth and mothers’ perception of partners’ warmth on the total behavioral problems and the internalizing and externalizing behavior of their children was studied. The percentage of congruence was 60%. In 19% of dyads, fathers reported higher warmth compared with how mothers rated them, and in 20% of dyads, fathers indicated lower warmth compared with mothers’ perception of their warmth. First, the model with total behavioral problems as an outcome variable was explored. The polynomial regression model was significant (*R*^2^ = 0.097, *p* < 0.001). The coefficients and the response surface suggested the presence of a significant linear additive effect on the line of congruence (*a*_1_ = 0.24, *p* < 0.001), indicating that the total behavioral problems of children were higher when fathers’ self-reported warmth and mothers’ perception of their warmth matched at higher levels (Figure 3). 

**Figure 4:**
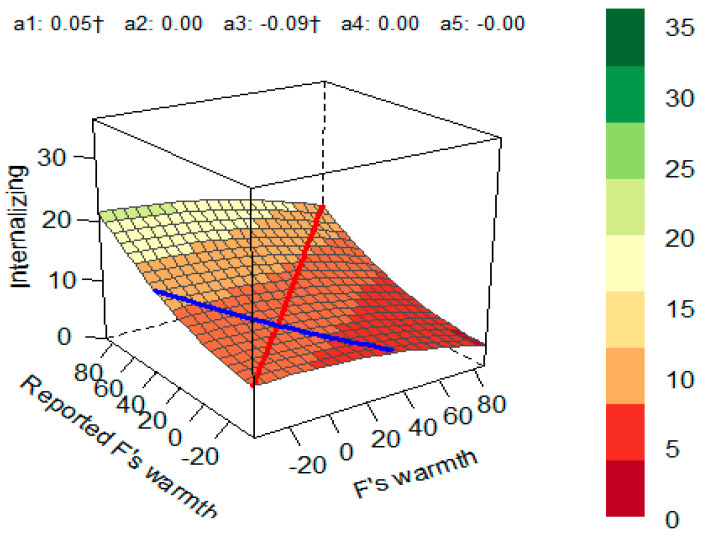
Response surface for the impact of fathers’ self-reported warmth and mothers’ perception of paternal warmth on internalizing problems in children. Red line—line of congruence; blue line—line of incongruence (LOIC); *a*_1_—slope symmetry line (linear additive effect on the line of congruence); *a*_2_—curvature symmetry line (curvature on the line of congruence); *a*_3_—slope asymmetry line (ridge shifted away from the LOC); *a*_4_—curvature asymmetry (line curvature on the line of incongruence) *a*_5_—the interaction coefficient between the first and second predictor variables. † *p* < 0.1.

A similar model using internalizing problems as an outcome variable also yielded significance (*R*^2^ = 0.039, *p* = 0.034). The coefficients and the response surface suggested the presence of trending effects, suggesting that children’s internalizing problems were higher when fathers’ self-reported warmth and mothers’ perception of their warmth matched at high levels, but none of them reached statistical significance (*a*_1_ = 0.05, *p* = 0.05). The slope asymmetry line for LOC was significant (*a*_3_ = −0.09, *p* = 0.05), pointing to the trend where internalizing problems were higher when fathers’ self-reports of warmth were higher than mothers’ perception of them compared with situations when mothers’ reports of fathers’ warmth were higher than the fathers’ self-reports (Figure 4). 

**Figure 5:**
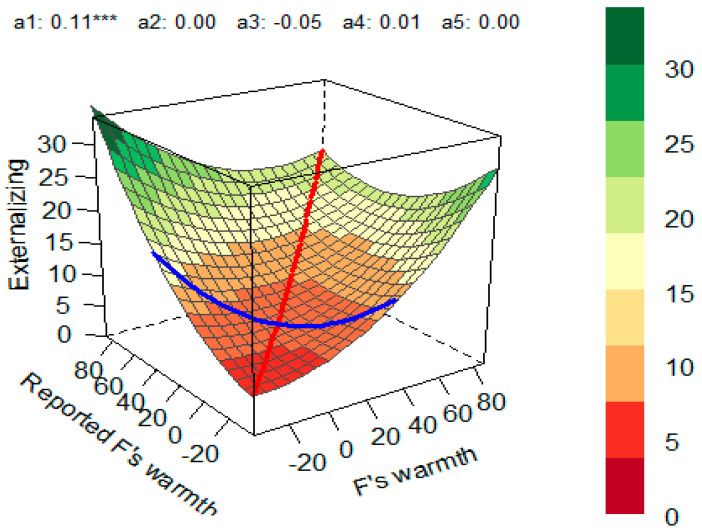
Response Surface for the Impact of Fathers’ Self-Reported Warmth and Mothers’ Perception of Paternal Warmth on Externalizing Problems in Children. Red line—line of congruence; blue line—line of incongruence (LOIC); *a*_1_—slope symmetry line (linear additive effect on the line of congruence); *a*_2_—curvature symmetry line (curvature on the line of congruence); *a*_3_—slope asymmetry line (ridge shifted away from the LOC); *a*_4_—curvature asymmetry (line curvature on the line of incongruence) *a*_5_—the interaction coefficient between the first and second predictor variables. *** *p* < 0.001.

A similar model using children’s externalizing problems as an outcome was significant (*R*^2^ = 0.127, *p* < 0.001). The additive effect on the line of congruence (*a*_1_ = 0.105, *p* < 0.001) indicated that matches between fathers’ self-report and mothers’ perception of fathers’ warmth at high values of the predictor had different outcomes compared with matches at the low values. Specifically, externalizing problems were higher when fathers’ self-report of warmth and mothers’ perception of fathers’ warmth matched at higher levels (Figure 5). 

Next, the dimension of demandingness was explored. The percentage of congruence was 60%. In 27% of dyads, mothers reported higher demandingness compared with fathers’ rating of them, and in 12% of dyads, mothers indicated lower demandingness compared with fathers’ perceptions of them. The congruence between mothers’ demandingness and the fathers’ report of maternal demandingness was evaluated in relation to children’s total behavioral problems score. The polynomial model was significant (*R*^2^ = 0.037, *p* = 0.044), and the coefficients suggested that children had a higher number of behavioral problems when mothers’ and fathers’ ratings of mothers’ demandingness matched at lower levels of demandingness (*a*_1_ = −0.178, *p* = 0.007). A model with the same predictors using internalizing behavior as an outcome did not yield any significance (*R*^2^ = 0.025, *p* = 0.076). However, the effect of the congruence was observed for the model using externalizing problems index as an outcome (*R*^2^ = 0.044, *p* = 0.019). The direction of the effects suggested that the match between parental reports on lower levels of maternal demandingness was associated with more externalizing problems in children (*a*_1_ = −0.06, *p* = 0.007). No effects for congruence between fathers’ demandingness and mothers’ reports of paternal demandingness were observed (*R*^2^ = 0.024, *p* = 0.197 for the model using total behavioral problems as an outcome; *R*^2^ = 0.021, *p* = 0.285 for internalizing; and *R*^2^ = 0.024, *p* = 0.191 for externalizing). 

## 4. Discussion

Parenting styles are associated with child mental health problems, academic achievement, adaptive functioning, delinquency, and interpersonal relationships [23], but little attention has been given to the mother–father differences in parenting styles within the family. The primary goal of the current study was to investigate the associations between self- and partner-perceived parenting styles of mothers and fathers and children’s mental health problems. We focused on a homogeneous sample of middle-class intact Russian families to expand the cross-cultural literature and to clarify the parental perceptions on childrearing in a culture that blends Western and Eastern values. 

The first goal of the current paper was to identify how parents perceived their own and their partners’ parenting styles. The differences between self-reports vs. partner reports on parenting are typically viewed as an auxiliary measure aimed at increasing validity [4]. Our study demonstrates the importance of accounting for differences in self-perceived vs. partner-perceived reports of parenting. The findings indicate that fathers had a higher agreement between self- and partner-perceived parenting than mothers. However, fathers underestimated both assessed dimensions of their parenting: demandingness and warmth compared to mothers’ reports.

Second, we aimed to identify distinct clusters of mental health problems in children based on parents’ reports and their relation to the discrepancy in parenting styles. Overall, our findings are in line with the literature showing that discrepancy in reports on parenting styles is associated with children’s mental health problems [3,56]. However, unlike other studies that mainly reported negative outcomes associated with the disagreement in parental reports, we found that fathers’ overestimation of their warmth relative to the mothers’ report was associated with a lower likelihood of being in a high severity profile of mental health problems in children. The differences in fathers’ and mothers’ perceptions of fathers’ demandingness were positively related to children’s mental health problems. The direction of the difference suggests that when fathers overestimated their demandingness compared with how mothers rated them, children were more likely to be classified into the group with moderate mental health problems. The findings indicate that not only does the overall discordance between how parents view themselves vs. how their partner views them affects the children’s mental health, but also the direction of the discrepancy plays a significant role. This finding aligns with the literature showing that an authoritarian style characterized by a combination of high demandingness and low warmth in fathers is linked to an increased rate and severity of internalizing and externalizing problems [20]. The issue of informant bias in assessing parenting styles is a significant methodological issue given that when a discrepancy between informants is encountered, it is difficult to determine whose report—self or partner’s—should serve as a reference point. Therefore, using a single partner’s report as a gold standard reference point might not provide an accurate evaluation of the trait. Even though we cannot directly conclude whose report would be more reliable, the main goal of the analysis was to assess the contribution of differences in self-reports vs. partner’s reports of parenting to the severity of children’s mental health problems. 

While the absolute difference between fathers’ self-perception of warmth and mothers’ perception was associated with a higher likelihood of children being classified in the high-severity group, fathers’ overestimation of their warmth relative to how mothers rated them was associated with a lower likelihood of being classified in the high severity group. The pattern of paternal overestimation of both demandingness and warmth compared with the mother’s report suggests a biased perception of their involvement in the childrearing process. Given that mothers are often considered primary caregivers, fathers may not give themselves enough credit for how much their expression of warmth and demandingness affects children. The importance of paternal involvement and warmth, in particular, has been demonstrated in a myriad of studies evaluating children’s social skills [17], self-efficacy [57], and academic outcomes [58].

Our findings indicate a common gender role assignment in the Russian culture, where fathers do not consider themselves active contributors to the childrearing process [59,60]. The dimensional approach to the assessment of parenting styles allowed us to see that not all discrepancies in self- vs. partner-perceived parenting were negatively linked to children’s mental health. In fact, the overestimation of the father’s demandingness but not the father’s warmth was linked to a higher severity of mental health problems in children. Given the small effect size, this finding needs to be interpreted with caution. 

Our results show that mothers tended to underestimate their warmth and overestimate their demandingness compared with their partners’ reports. While the findings are anchored to the partners’ reports, mothers tended to see themselves as more authoritarian than their partners perceived them. While the authoritarian parenting style has a negative connotation in Western cultures, in other parts of the world, it can be seen as an effective parenting approach. A meta-analysis showed that in Arab countries and Eastern Europe, authoritarian parenting was not associated with any negative outcomes in children [25]. In Asian cultures, authoritarian parenting is linked to higher academic outcomes [61]. Research on ethnic minorities in the US shows that high control and close supervision are linked to lower delinquency in children [62]. Given that an authoritarian style is not necessarily a negative strategy, as initial research suggested, we can offer three potential explanations for why mothers tended to overestimate their demandingness and underestimate their warmth. First, self-reports might be driven by the social desirability of the answers. In Russian culture, showing too much warmth can be seen as a sign of spoiling the child, while the expression of high demandingness and control is typically associated with effective discipline. Therefore, mothers might try to depict themselves as more authoritarian than they are. The second potential explanation is that mothers try to act in a more authoritarian way in accordance with their values; however, the fathers perceive them as more authoritative than authoritarian. Finally, mothers might underestimate warmth and overestimate demandingness while still being relatively low on these traits, as mothers’ perception is anchored to partners’ reports.

The traditional analysis was complemented with an RSA approach to explore how the degree of congruence and incongruence between parental reports affected children’s mental health problems. The results obtained using this approach went beyond the traditional difference scores, as they provided information on the directionality, extent, and importance of congruence for the outcomes of interest. The effect of maternal warmth was a robust predictor, showing that children whose parents agreed on high maternal warmth exhibited more total behavioral problems qualified by externalizing. Previous research suggests that maternal warmth can be a protective factor against maladjustment in the presence of adversity [63]; however, excessive demonstration of warmth is typically linked to permissive parenting. It was shown that the maternal permissive parenting style uniquely explains 44% of the variance in children’s externalizing behaviors [18]. 

The incongruence between fathers’ self-reported warmth and mothers’ report of fathers’ warmth was significant in predicting children’s internalizing problems in this study: higher internalizing in children was associated with a pattern when fathers’ self-report of warmth was higher than mothers’ perception of fathers’ warmth. This suggests that unrealistic self-reflection of fathers’ warmth (i.e., overestimation) is linked to internalizing in children, including depression and anxiety. This finding can have two potential explanations. First, fathers may be over-reporting their warmth due to social desirability [64]. Alternatively, incongruence between fathers’ self-report and mothers’ perception can be indicative of fathers’ lower emotional intelligence, which could signify lower emotional availability than they believe they show. Paternal emotional availability is an important predictor of children’s well-being [65] and emotional competence [66]. Inaccurate self-perception in the context of caregiving was previously shown to be a risk factor for developing behavioral problems in children; however, there are biases in the perceptions of both parents [67]; therefore, the interpretation of the origins of discrepancy are speculative. Finally, the results of RSA suggested that children tended to have a higher number of behavioral problems when mothers’ and fathers’ ratings of mothers’ demandingness matched at lower levels of demandingness. This finding points to a permissive parenting style being a risk factor, which is in line with the previous body of literature [18,68]. The lack of maternal but not paternal demandingness (corroborated by self-reports and partner reports) was likely predictive of behavioral problems due to mothers’ traditional role of a primary caregiver [69]. 

Taken together, the results obtained using the RSA approach suggest that both congruence and incongruence between parenting styles could be predictive of children’s behavioral problems. In particular, when parents agreed about high warmth and matched on lower levels of demandingness, in line with the permissive parenting style, children tended to exhibit maladaptive behavior and externalizing problems. Most of the outcomes that were associated with behavioral problems were driven by externalizing problems. Limitations of this approach include the inability to perform multivariate analysis; therefore, warmth and demandingness were viewed separately. 

The current study had several limitations. First, it did not include children’s reports of parenting styles. Studies show that parent–child agreement on parenting styles is small to moderate depending on the children’s age [70]. It was shown that parents tended to underreport problematic parenting strategies while children overreported them [71]. While our study focused broadly on dimensions of parenting styles, it is possible that by providing only the parent’s perspective, we are overlooking an important source of information. Future studies should aim to incorporate children’s, parents’, and teacher’s reports of children’s mental health and well-being to provide a more holistic view of children’s functioning. Given the findings of the current study, cross-informant agreement could serve not only as a tool to increase the validity but also as clinically relevant information on contextual factors in children’s behavioral and emotional problems. A second limitation of the study was the inability to assess children’s mental health problems through their own reports. Integrating parents’, children’s, and teacher’s reports in the future studies could guide intervention planning. When all parties agree on specific behavioral concerns, targeted interventions can be designed to address those challenges more effectively. Integrating the reports of multiple informants would enhance the comprehensiveness and validity of the assessments, assist in the identification of risk and protective factors, and aid in intervention planning. Third, we only analyzed the data from intact heterosexual families, leaving open the question about the generalizability of the findings to more diverse family structures. Future studies should incorporate collateral reports of mothers’ and fathers’ parenting from children and people outside of the nuclear family system to increase objectivity. Additionally, the current study focused on the dimensions of perceived parenting styles not taking into consideration specific rule setting within the parenting practices. Rules, as part of behavioral strategies, are closely linked to the dimension of demandingness and can significantly influence children’s behavior and outcomes. Previous research indicated that effective parental rule setting can act as a protective factor against certain externalizing behaviors, including substance use [72]. The current study primarily focused on self- and partner-perceived parenting styles, which encompass broader aspects of parenting and may not provide a comprehensive understanding of the role of rule-setting in shaping children’s behaviors. Future research could consider incorporating a more nuanced assessment of parenting practices, including rule-setting, to gain a more comprehensive understanding of its impact on children’s outcomes. Finally, the data that informed the findings of this manuscript were collected between the years 1999 and 2000; therefore, it might not capture the evolving dynamics of parenting behaviors in Russia. Therefore, societal shifts and cultural changes that have occurred in the country since the data collection might affect the generalizability of the findings to present-day parenting practices. 

Our study expands the existing literature by providing a cross-cultural perspective on differences in parenting styles, viewing the construct dimensionally instead of assigning discrete categories, and by identifying latent profiles of mental health problems in children as they relate to discrepancies in parenting styles. The dimensional approach allowed us (1) to assess the full spectrum of parenting styles without constraining them to specific categories, which are often not mutually exclusive, and (2) to account for the comorbidity of mental health problems in children. Our findings might inform clinical work with families by emphasizing the role of fathers’ involvement and its impact on children’s well-being. Based on the study’s results, several policy recommendations can be proposed to support children’s mental health and well-being. First, there is a need for parenting interventions and programs that emphasize the importance of consistent parenting styles within families. Providing resources and guidance to parents on effective communication and cooperation in parenting (ranging from parental psychoeducation to parent management training) can help reduce incongruences and promote positive mental health outcomes for children. Second, recognizing the significance of parental warmth in children’s mental health, policies should encourage fathers’ active involvement in caregiving and emotional support. Initiatives aimed at promoting positive father–child interactions and dispelling traditional gender stereotypes can foster a nurturing family environment and positively impact children’s mental health. Third, the study highlights the importance of addressing maladaptive behaviors and externalizing problems associated with permissive parenting styles. Developing targeted interventions for families that are characterized by such parenting practices can assist in mitigating the negative effects of permissive parenting on children’s mental health.

## 5. Conclusions

This study aimed to identify latent profiles in children’s mental health problems in relation to the magnitude and direction of (dis)agreements between self-reported and partner-reported parenting styles in Russian families. The results show that the discrepancy between self-perceived and partner reports of parenting styles could serve as a significant predictor of mental health problems in children. The dimensional approach to the assessment of parenting styles allowed us to observe that not all discrepancies in self- vs. partner-perceived parenting were negatively linked to children’s outcomes. In fact, the inaccurate perception of the fathers’ demandingness but not the fathers’ warmth was linked to a higher severity of mental health problems in children. Future studies should further investigate how paternal appraisal of parenting relates to other areas of children’s functioning, including adaptive functioning and academic achievement. Future work should aim to obtain collateral reports of parenting styles to improve objectivity and to include families of diverse structures (e.g., single parents, same-sex parents) and ethnic backgrounds.

## Figures and Tables

**Table 1:** Means, Standard Deviations, and Correlations between Parenting Styles.

Variable	*M*	*SD*	1	2	3	4	5	6	7	8	9	10	11
1. |MW − FPW|	9.94	11.16											
2. |FW − MPW|	10.69	12.37	0.48 **										
3. |MD − FPD|	9.96	11.12	0.31 **	0.28 **									
4. |FD − MPD|	10.08	10.66	0.24 **	0.37 **	0.56 **								
5. |MW − FW|	12.95	11.65	0.45 **	0.39 **	0.15 **	0.23 **							
6. |MD − FD|	13.29	12.32	0.16 **	0.24 **	0.62 **	0.61 **	0.26 **						
7. MW − FPW	−4.38	14.30	–0.48 **	–0.07	–0.00	–0.08	–0.26 **	0.01					
8. FW − MPW	−1.79	16.26	0.05	–0.42 **	–0.15 *	–0.10	0.20 **	–0.10	–0.39 **				
9. MD − FPD	3.64	14.48	0.07	0.09	0.43 **	0.12 *	–0.00	0.33 **	0.20 **	–0.18 **			
10. FD − MPD	−0.81	14.66	0.01	–0.09	–0.24 **	–0.03	–0.02	–0.28 **	–0.19 **	0.12 *	–0.66 **		
11. MW − FW	−6.09	16.33	–0.19 **	0.10	0.08	–0.04	–0.51 **	0.02	0.60 **	–0.65 **	0.24 **	–0.20 **	
12. MD − FD	5.93	17.14	0.02	0.14 *	0.28 **	0.10	0.12 *	0.44 **	0.15 **	–0.20 **	0.74 **	–0.76 **	0.15 **

*Note:* MW—mother’s self-reported warmth, MD—mother’s self-reported demandingness, MPW—mother’s perception of her partner’s warmth, MPD—mother’s perception of her partner’s demandingness, FW—father’s self-reported warmth, FD—father’s self-reported demandingness, FPW—father’s perception of his partner’s warmth, FPD—father’s perception of his partner’s demandingness, *M*—mean, and *SD*—standard deviation. * *p* < 0.05. ** *p* < 0.01.

**Table 2:** LPA Model Fit Summary for the CBCL Scores.

Class	AIC	BIC	SABIC	Entropy	Smallest Class %	VLMR-LRT *p*-Value	VLMR-LRT Meaning	BLRT *p*-Value	BLRT Meaning
1	18,177.58	18,237.10	18,186.36	-	-	-	-	-	-
2	17,343.68	17,436.68	17,357.40	0.94	30%	0.00	2 > 1	0.00	2 > 1
3	17,197.98	17,324.47	17,216.64	0.83	21%	0.04	3 > 2	0.00	3 > 2
4	17,061.84	17,221.81	17,085.44	0.87	5%	0.26	4 = 3	0.00	4 > 3

*Note:* AIC—Akaike information criterion; BIC—Bayesian information criterion; SABIC—sample-size adjusted BIC; VLMR-LRT—Vuong–Lo–Mendell–Rubin adjusted likelihood ratio test; BLRT—bootstrapped likelihood ratio test; CBCL—Child Behavior Checklist.

**Table 3:** Predictors of Membership in the Latent CBCL Profile.

	Moderate						High					
Model	1	2	3	4	5	6	1	2	3	4	5	6
Gender	1.23	1.22	1.27	1.23	1.25	1.25	0.48 *	0.48 *	0.50 *	0.50 *	0.49 *	0.48 *
Age	0.90	0.91	0.91	0.91	0.91	0.91	0.85 **	0.85 **	0.86 **	0.85 **	0.86 *	0.85 **
|FW − MPW|	1.02						1.04 **					
|FD − MPD|	0.99						0.98					
FW − MPW		0.99						0.96 **				
FD − MPD		1.04 **						1.01				
|MW − FPW|			1.01						1.01			
|MD − FPD|			1.01						1.01			
MW − FPW				0.99						1.01		
MD − FPD				0.99						1.02		
|MW − FW|					1.02						1.03	
|MD − FD|					1.01						0.99	
MW − FW						1.00						1.01
MD − FD						0.99						1.00

*Note:* MW—mother’s self-reported warmth, MD—mother’s self-reported demandingness, MPW—mother’s perception of her partner’s warmth, MPD—mother’s perception of her partner’s demandingness, FW—father’s self-reported warmth, FD—father’s self-reported demandingness, FPW—father’s perception of his partner’s warmth, FPD—father’s perception of his partner’s demandingness, *M*—mean, and *SD*—standard deviation. * *p* < 0.05. ** *p* < 0.01.

## Data Availability

The datasets generated and/or analyzed during the current study are available in the OSF repository: https://osf.io/gr3p4/?view_only=03d24e91c020426995c6fccf0c2531fe (accessed on 3 August 2023).
